# Supplementary material for: Identification of photoperiod-induced specific miRNAs in the adrenal glands of Sunite sheep (Ovis aries)
Source: Front Vet Sci. 2022 Jul 22;9:888207. doi: 10.3389/fvets.2022.888207 (PMC9354845; doi:10.3389/fvets.2022.888207)
Supplement: Supplementary file 1 [file Data_Sheet_1.ZIP › Supplementary file/miRDeep2 file.zip!/miRDeep2 file/Know_Structure/SPLP42A1/oar-mir-103.pdf]

miRBase precursor : oar-mir-103
Total read count : 16192
oar-miR-103 read count : 16192
remaining reads : 0

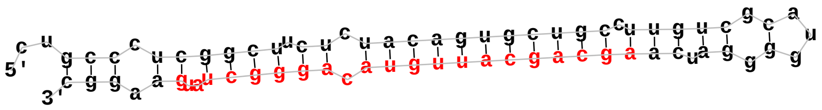

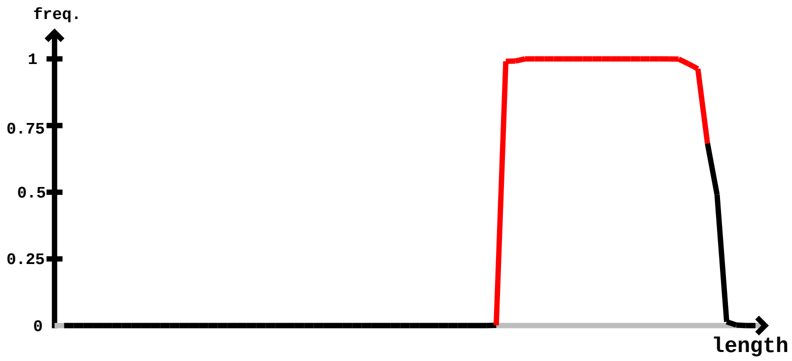


```
                                               oar-miR-103
5'- cugcccucggcuucucuacagugcugccuugucgcauggggaucaagcagcauuguacagggcuaugaaggc   -3'    exp
    ..(((.((((((.((.(((((((((((.(((((.(...).)).))))))))))))))).))))))..)).)))    reads      mm         sample
    ............................................aagcagcauuguacagggcuau......    1          0          seq
    ............................................aagcagcauuguacagggcuaug.....    5          0          seq
    ............................................aagcagcauuguacagggcuauga....    11         0          seq
    .............................................agcagcauuguacaggg..........    3          0          seq
    .............................................agcagcauuguacagggc.........    10         0          seq
    .............................................agcagcauuguacagggcu........    280        0          seq
    .............................................agcagcauuguacagggcua.......    312        0          seq
    .............................................agcagcauuguacagggcuau......    4516       0          seq
    .............................................agcagcauuguacagggcuaug.....    3086       0          seq
    .............................................agcagcauuguacagggcuauga....    7608       0          seq
    .............................................agcagcauuguacagggcuaugaa...    184        0          seq
    .............................................agcagcauuguacagggcuaugaaA..    24         1          seq
    .............................................agcagcauuguacagggcuaugaag..    5          0          seq
    ..............................................gcagcauuguacagggcu........    1          0          seq
    ..............................................gcagcauuguacagggcuau......    3          0          seq
    ..............................................gcagcauuguacagggcuaug.....    2          0          seq
    ..............................................gcagcauuguacagggcuauga....    11         0          seq
    ..............................................gcagcauuguacagggcuaugaa...    1          0          seq
    ...............................................cagcauuguacagggcuau......    6          0          seq
    ...............................................cagcauuguacagggcuaug.....    16         0          seq
    ...............................................cagcauuguacagggcuauga....    91         0          seq
    ...............................................cagcauuguacagggcuaugaa...    10         0          seq
    ...............................................cagcauuguacagggcuaugaaA..    3          1          seq
    ...............................................cagcauuguacagggcuaugaag..    1          0          seq
    ................................................agcauuguacagggcuauga....    1          0          seq
    ................................................agcauuguacagggcuaugaa...    1          0          seq
```
